# Frequency Tuning in the Behaving Mouse: Different Bandwidths for Discrimination and Generalization

**DOI:** 10.1371/journal.pone.0091676

**Published:** 2014-03-14

**Authors:** Livia de Hoz, Israel Nelken

**Affiliations:** 1 Department of Neurobiology, the Silberman Institute for Life Sciences, and the Edmond and Lily Safra Center for Brain Sciences. Hebrew University of Jerusalem, Jerusalem, Israel; 2 Max Planck Institute for Experimental Medicine, Göttingen, Germany; Rutgers University, United States of America

## Abstract

When faced with sensory stimuli, an organism may be required to detect very small differences in a physical parameter (discrimination), while in other situations it may have to generalize over many possible values of the same physical parameter. This decision may be based both on learned information and on sensory aspects of perception. In the present study we describe frequency processing in the behaving mouse using both discrimination and generalization as two key aspects of behaviour. We used a novel naturalistic behavioural apparatus designed for mice, the Audiobox, and paradigm contingencies that were identical for both auditory discrimination and generalization, the latter measured using latent inhibition. Mice learned to discriminate between frequencies that were an octave apart in a single trial. They showed significant discrimination between tone frequencies that were as close as 4–7%, and had d' of about 1 for ΔF of around 10%. In contrast, pre-exposure frequencies that were half an octave or less below the conditioned tone elicited latent inhibition, showing a generalization bandwidth of at least half an octave. Thus, in the same apparatus and using the same general memory paradigm, mice showed generalization gradients that were considerably wider than their discrimination threshold, indicating that environmental requirements and previous experience can determine whether the same two frequencies will be considered same or different. Remarkably, generalization gradients paralleled the typical bandwidths established in the auditory periphery and midbrain, suggesting that frequencies may be considered similar when falling within the same critical band.

## Introduction

Two sensory stimuli may be highly discriminable, and yet sufficiently similar to be considered essentially the same. In natural speech, for example, two utterances of the same word by the same speaker, even with the same nominal pitch trajectory, would almost certainly be physically different and (with some effort) also discriminable from each other; nevertheless, as a rule, they would be judged ‘the same’. In natural contexts, both discrimination and generalization may have important roles. While discrimination is generally believed to be limited by peripheral sensory factors, generalization may be driven by context and task requirements [1]. Nevertheless, it is reasonable to hypothesize that animals have formed, even without explicit training, some ideas about what stimuli should or should not be considered the same. In the present study we set out to describe frequency-driven behaviour in the mouse using both discrimination and generalization as two key aspects of perception.

Mice have good acoustic abilities. Audiograms, as well as thresholds for frequency or duration discrimination, are well described in the rodent literature using a variety of tasks [2], training procedures [3]–[4], ages [5] (Jackson laboratories), developmental experience [6], and strains (Jackson laboratories). Mice discriminate between frequencies that are separated by 3% or less when detecting the frequency difference between one stimulus and the next [7]–[8]. Less is known, however, about the abilities of mice to make absolute comparisons between two learned stimuli that are presented separately in time.

Little is known about the capacity of mice to generalize across stimuli. We studied generalization using latent inhibition. Latent inhibition refers to the diminished capacity of a conditioned stimulus to be associated with reinforcement when that stimulus has been previously presented to the subject in a non-reinforced manner [9]. Stimuli that are similar to the conditioned stimulus can also lead to latent inhibition due to a generalization over some stimulus property, such as the frequency of a tone (e.g. [10]). We chose the latent inhibition paradigm since the task itself does not determine the level of generalization [1]. Measurements of generalization gradients using conditioned responses have been made [10], but to the best of our knowledge ours is the first attempt to use latent inhibition in mice for that purpose.

Here we used the Audiobox, where training occurs automatically and the animals perform the task *ad libitum*. This paradigm is fully automatic, allowing both continuous recording of individual mouse behaviour in its living quarters, as well as the training and monitoring of several cohabiting mice. In consequence, we could train mice relatively quickly to discriminate between two frequencies and we could reliably measure generalization gradients using latent inhibition. The main result of this paper is the measurement, in the same apparatus and using the same technique, of small frequency discrimination thresholds (of few per cents) and much wider generalization gradients (spanning at least half an octave).

## Methods

Ethics statement: Experiments were performed in The Hebrew University of Jerusalem and in the Max Planck Institute for Experimental Medicine in Göttingen. The joint ethics committee (IACUC) of the Hebrew University and Hadassah Medical Center approved the study protocol for animal welfare. The Hebrew University is an AAALAC International accredited institute. All animal experiments that took place in Göttingen were approved by and performed in accordance with the Niedersächsisches Landesamt für Verbraucherschutz und Lebensmittelsicherheit, project license number 33.14-42502-04-10/0288.

The main set of experiments was performed in Jerusalem. A replication of key subset of experiments was done in Göttingen, to check the reproducibility of data with this new apparatus. Some variations, such as roving of stimulus intensities, were introduced in Göttingen, and did not affect the results. We therefore report the results of the main experiments run in Jerusalem in the paper. The details of the replications in Göttingen are reported in the Text S1 and Figures S1 and S3.

We used 135 C57BL/6JOlaHsd female mice obtained from a commercial supplier (98 from Harlan, Israel; 35 from Harlan, Germany). The mice were 7–8 weeks (Israel) and 5–6 weeks (Germany) old at the time of arrival and were always kept in a light/dark 7am/7pm cycle. A few days after arrival, or 4 weeks after arrival in the group that began training with 9–10 weeks of age, each mouse was lightly anaesthetized with isoflurane vapour or avertin i.p. and a sterile transponder (DATAMARS T-IS 8010 FDX-B, 13 mm long, 2 mm in diameter, 0.1 gr in weight; or IS0 compliant 11784 transponder, 12 mm long, from TSE) was implanted subcutaneously in the upper back. In later replications, a stitch or histoacryl (Braun) was used to close the small hole left on the skin by the transponder injection. Once recovered from anaesthesia, the mice were placed in the Audiobox (see below).

### Apparatus: Audiobox

All behaviour was run in an Audiobox (New Behaviour/TSE, Germany), a device developed for auditory research and based on the Intellicage (NewBehavior, Switzerland). The model used in Jerusalem was a prototype, while the one used in Göttingen is commercially available (TSE). The Audiobox serves both as living quarters for the mice and as their testing arena. The mice are kept in groups of 8 to 10 animals. Each animal is individually identifiable through the use of the implanted transponder, and the behaviour of each mouse is automatically detected by two means: reading of the unique transponder carried by each mouse by an antenna at the entrance to the drinking corner (see below), and detection of specific behaviours (nose-poking and licking) through other sensors. In consequence, handling of the animals by the experimenter is reduced to the weekly cleaning of the cages and apparatus.

**Figure 1:**
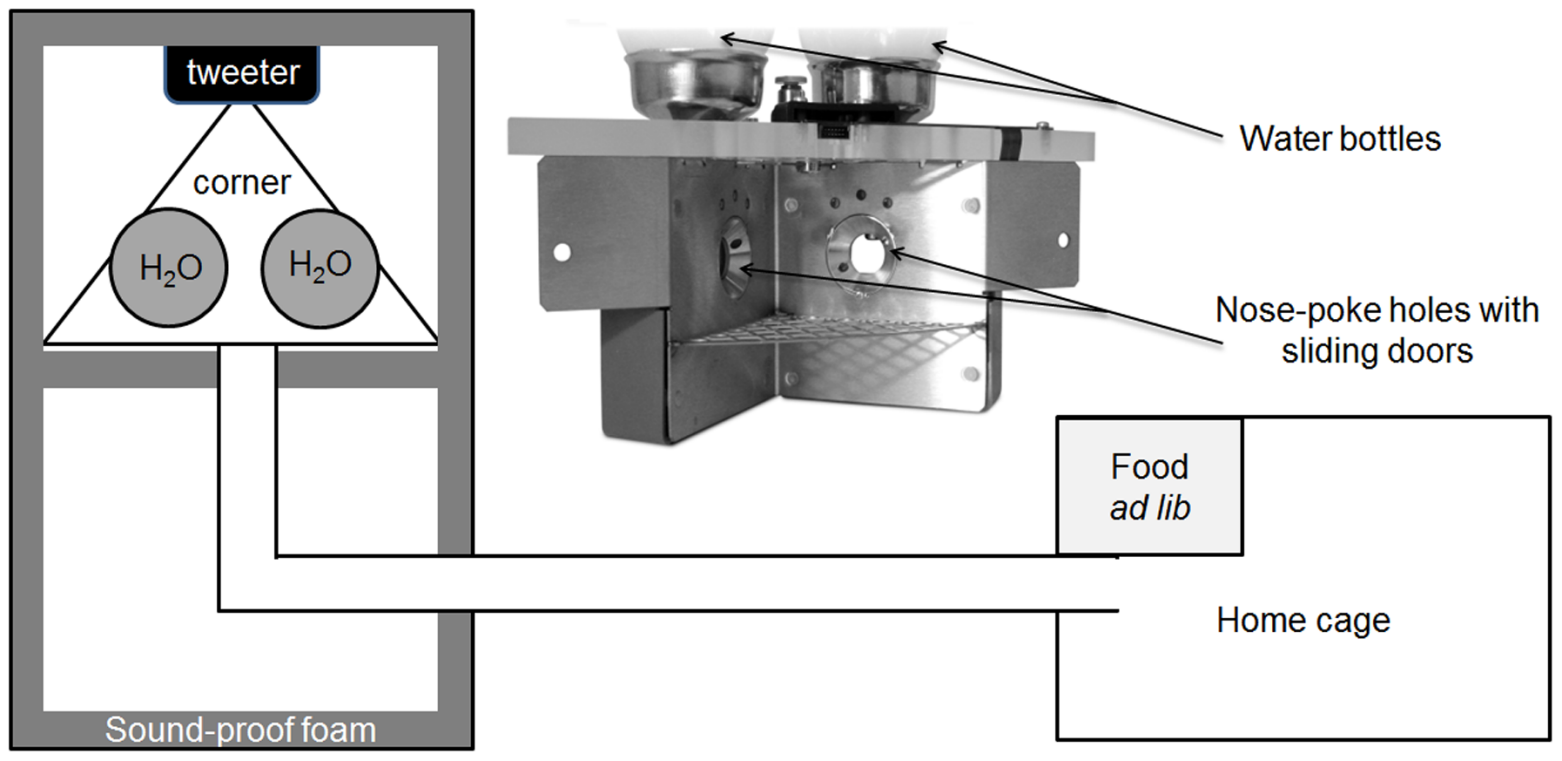
Apparatus. Schematic of the Audiobox and (insert) photo of the corner (from www.tse-systems.com, with permission). The attenuated box in divided in two chambers, one contains the corner with the two bottles of water and the tweeter behind. A corridor connects this box to the home-cage where food is available *ad libitum*.

The Audiobox was kept in a dedicated room, used only for these experiments (with no other animals present), temperature regulated and kept in a 12 hr/12 hr dark/light cycle. The Audiobox consists of two compartments connected by a long corridor (Figure 1). One compartment, a normal mouse cage, serves as the home cage, where the animals have access to food *ad libitum*. Water is delivered in the second compartment of the Audiobox, the ‘corner’ (see image insert in Figure 1), which is positioned inside a sound-attenuated box. Entrance into the corner, a ‘visit’, is detected by an antenna located at its opening that reads the implanted transponder. The beginning of the visit is defined by both the detection of a transponder by the antenna and the activation of a heat sensor within the corner. The end of the visit occurs when the same transponder is not detected anymore by the antenna and the heat sensor is no longer activated. Thus, the Audiobox identifies the specific mouse that enters the corner, and can therefore select the stimulus to be presented accordingly. All behavioural data is logged for each mouse individually. Once in the corner, the mouse can access water by nose-poking, a ‘nose-poke’, into either of two ports, one at each side of the corner. The doors to the ports can be opened or closed depending on the demands of the experiment. A loudspeaker (DSM 25 FFL 8 Ohm from Visaton, or a 22TAF/G from Seas Prestige) is positioned directly behind the corner, or above it, for the presentation of the stimuli.

We used mainly tones between 6 and 15 kHz, and for some experiments between 3 and 19 kHz. This frequency range contains the area of highest sensitivity in a mouse audiogram. Natural background sounds, which the mouse might use for information and warning, tend to be within this frequency range. While some mouse vocalizations have frequencies above 45 kHz, other mouse communication calls also occupy this range: for example, wriggling calls emitted by pups contain mostly frequencies below 20 kHz [11].

Sounds were generated using Matlab (Mathworks) at a sampling rate of 96 or 48 kHz and written into computer files. Output was calibrated using either a Brüel and Kjaer (4939 ¼″ free field) or a GRAS (1/4″ 40BE) microphone. The microphone was placed at different positions within the corner, as well as outside the corner. Relevant sounds were played at the nominal intensities used in the study. Microphone signals were sampled at 96 kHz and analyzed in Matlab. Tones between 3 kHz and 19 kHz did not show any significant harmonic distortion. In the rare occasions when harmonics were present, they were at least 40 dB below the main signal. There was a linear correspondence between the nominal sound level and the sound level measured by the microphone.

While sounds played inside the corner were significantly attenuated outside of the attenuated box (>20 dB), there was little attenuation between the corner and the corridor directly leading to it (about 10 dB). In consequence, mice in the corridor could hear the sound presented to the mouse inside the corner. This, however, did not seem to affect their behaviour. For example, animals that were pre-exposed to 13 kHz and then conditioned to the same frequency were run in several replications together with other mice that were pre-exposed to other frequencies. The amount of latent inhibition observed in the mice pre-exposed to 13 kHz was the same irrespective of the pre-exposure frequencies that cohabiting mice heard.

Stimuli consisted of 30 ms pure tone pips, with 5 ms rise/fall linear slopes, repeated at a rate of 3 Hz. Tones were presented in the corner throughout the visit. All tone pips presented within a given visit had the same frequency. For the main experiments (in Jerusalem), sounds were played at a fixed intensity of 70 dB SPL. For the replications in Göttingen, we used a roving-intensity paradigm (See Text S1).

Throughout the duration of the experiment, one frequency (i.e. 6670 Hz) was always ‘safe’: when this frequency was presented during a visit, the mice could access the ports and drink water without an associated negative outcome. At some point within the training, a different tone frequency was associated with an air puff. The purpose of the training was to get the mice to learn this association.

### Discrimination

**Figure 2:**
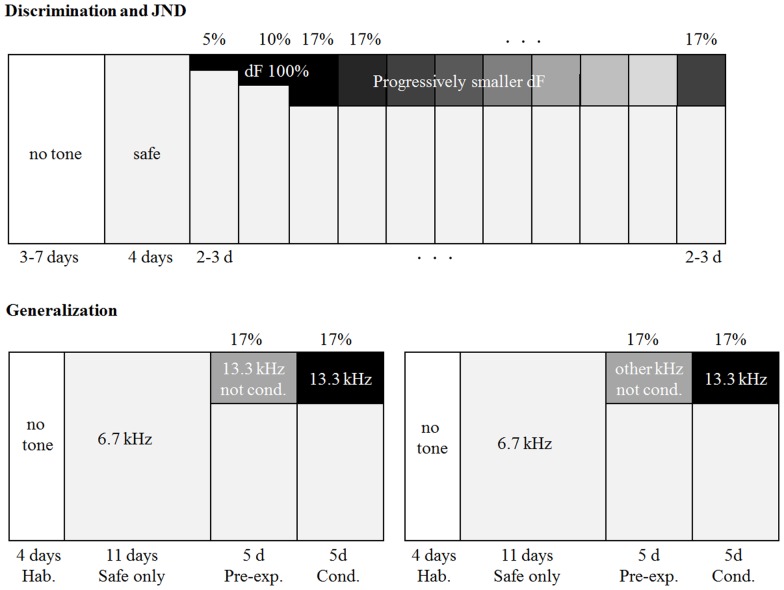
Paradigm. A. Schematic of the discrimination training protocol. The horizontal axis of the box represents time and the vertical axis represents the percentage of visits that were safe (white) or conditioned (black to light grey). B. Left: schematic of the training protocol of the latent-inhibition group, pre-exposed to 13340 Hz and conditioned to 13340 Hz. Right: same, for the groups pre-exposed to a frequency that was different 13340 Hz.

The aim of this experiment was to train mice to discriminate between tones of two different frequencies. Initially, these were two well separated tones (1 octave apart). We then explored the capacity of the mice to discriminate between progressively more similar tones, by moving the conditioned tone closer in frequency to the safe tone, in order to establish the just-noticeable difference (JND). We ran 3 replications of the discrimination task. The first one was run in Jerusalem using 9 mice, 7–8 weeks old at the time of testing, that heard a 6670 Hz as safe tone and 13340 Hz as conditioned tone. The other two were run in Goettingen (See Text S1). The choice of frequencies was based on ABR threshold measurements (Jackson Laboratories phenotype database) which showed large increase in thresholds between 16 and 32 kHz. Therefore, we avoided using frequencies above 16 kHz. The detailed protocol was as follows (see Figure 2):

Habituation phase (7 days): Immediately after the transponder implantation, mice were placed into the Audiobox. During this phase the doors giving access to the water within the corner remained constantly open and no sound was presented during the visits.

Safe-only phase (4 days): Once the mice had learned to access the water and were drinking freely, the doors were closed and only opened when the mouse nose-poked into the port. At the same time, every visit to the corner was coupled, for the duration of the visit, with the presentation of the safe sound (tone pips).

Introduction of a conditioned tone at 5% probability (3 days): The conditioned tone was now presented in 5% of the visits. These visits will be termed ‘conditioned visits’. They occurred on the 11^th^ visit out of every 20. Mice didn't seem to predict the appearance of a conditioned visit in Jerusalem, as reflected in the lack of change in the behaviour in the safe visits around conditioned visits. As in the safe-only phase, a train of tone pips was presented during the visit. The presence of the conditioned tone had a behavioural significance: a nose-poke during the presentation of the conditioned tone resulted in the delivery of an aversive air-puff, through a tube located on the ceiling of the corner, and the doors that gave access to the water did not open. The remaining visits were as during the safe-only phase.

Conditioned tone at 10% probability (3–5 days): The conditioned tone was played in 10% of the visits. They occurred on the 6^th^ visit out of every 10.

Conditioned tone at 17% probability (3 days): The conditioned tone was played in 17% of the visits (randomly once every 6 visits).

Once discrimination at ΔF of 100% was stable with 17% conditioned visits, we began to lower the frequency of the conditioned tone. The safe tone remained constant in 83% of visits. The conditioned tone was reduced every two to three days from 13340 Hz (1 octave, ΔF = 100%), progressively down to 6803 (ΔF = 2%) in the first experiment run in Jerusalem (see Table 1 below for detailed description of all JND tones used). Finally, the frequency of the conditioned tone was brought back up to a ΔF of 20% to ensure that the mice remained under stimulus control.

**Table 1:** Sequence of conditioned frequencies used for JND assessment.

	Conditioned tone (% w respect to safe, Hz)							
Safe tone (Hz)	Δf 100%	Δf 40%	Δf 20%	Δf 15%	Δf 10%	Δf 7%	Δf 4%	Δf 2%
6670	13340	9433	8004	7670	7337	7137	6937	6803

### Generalization gradients for frequency

The aim of this experiment was to establish generalization gradients for pure tones in the mouse using the latent inhibition paradigm. For this purpose, different mice were pre-exposed to frequencies that were different, similar or identical to the subsequent conditioned frequency. After the pre-exposure ended all mice were conditioned to the same conditioned frequency and latent inhibition was measured as a function of the pre-exposure frequency heard. Latent inhibition was quantified by the reduction of the efficacy of conditioning during the first few conditioned tone presentations, before the mice learned to reliably avoid nose-poking during presentations of the conditioned frequency.

In total, 89 naïve mice were used for the generalization paradigm. Transponder implantation, habituation (4 days), and safe-only phase (11 days), were run as described for the 2-tone discrimination. Following the safe-only phase, training consisted of the following stages (see Figure 2):

Pre-exposure phase (5 days): The pre-exposed tone was now played in 17% of the visits (randomly once every 6 visits). This tone did not have behavioural significance and a nose-poke in these visits resulted, like in the safe visits, in the opening of the doors and access to water. The remaining 83% visits were associated with the safe tone as before.

The pre-exposure tone had one of the following frequencies: 3335 Hz (2 octaves below 13340 Hz, 9 mice), 5609 (1.25 octaves below, 4 mice), 7932 Hz (0.75 octave below, 8 mice), 9433 Hz (0.5 octave below, 14 mice), 11218 Hz (0.25 octave below, 16 mice), 13340 Hz (equal to the conditioned tone, 31 mice), or 15864 Hz (0.25 octave above, 7 mice). Generally 2 or 3 groups of pre-exposure visits were run simultaneously, and often one of these groups was the group pre-exposed to the frequency that was conditioned later, 13340 Hz.

Conditioned phase (5 days): This phase was identical for all mice, irrespective of their pre-exposure frequency. The pre-exposure visits were replaced by conditioned visits, also with a probability of 17%. For all mice, the conditioned tone had a frequency of 13340 Hz. During this phase, the conditioning tone had a behavioural significance: a nose-poke resulted in the delivery of an air-puff and the doors were not opened. The remaining 83% of the visits were safe visits. All the mice used in this report had at least 24 hours of conditioning.

### Quantifying performance: d' and C

Discrimination performance was quantified by the standard measures from signal detection theory, the discriminability (d') and the Criterion (C) [12]. Both refer to a hypothetical internal decision variable that determines whether the mouse should nose-poke or not. Conceptually, this decision variable is large when the mouse has a good indication that it should not nose-poke, and is small otherwise. Thus, the decision variable is assumed to have a larger mean when the conditioned tone is played than when the safe tone is played, and to have a Gaussian distribution around the corresponding mean with the same variance for both tones. Under these assumptions, d' is a measure of the distance between the means of the decision variable at presentations of the conditioned sound and safe sounds, in units of the common standard deviation. It is calculated as
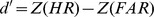
where Z(p), 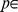
[0 1], is the inverse of the cumulative Gaussian distribution, HR is the hit rate, where a hit is the correct avoidance of a nose-poke in a conditioned visit, and FAR is the false alarm rate, where a false alarm is the avoidance of a nose-poke in a safe visit. C is the ‘threshold’: the value of the decision variable above which the animal avoids nose-pokes, measured in units of the common standard deviation of the decision variable, with C = 0 corresponding to the mid-point between the two means. C is a measure of the strategy used for the discrimination and its optimal value is affected by the costs of both misses and false alarms. With the same d', a mouse might prefer to be conservative, and to miss more safe visits rather than incorrectly nose-poke in a conditioned visit, or to be less conservative and drink more in safe visits, taking the risk that it would make more errors on conditioned visits. C = 0 corresponds to an observer that gives equal weights to misses and to false alarms. C is calculated as
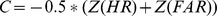



Negative values of C indicate that the mouse is more conservative (in our case, nose-poking less often on both safe and conditioned visits) than the unbiased observer, while positive values indicate that it is less conservative.

Since both d' and C cannot be calculated when either the hits or the false alarms reach levels of 100% or 0%, in the few cases where this happened we used 95% and 5% respectively for these calculations. This manipulation reduced d' slightly, and therefore our d' estimates are conservative.

## Results

Using a novel behavioural apparatus, the Audiobox (New Behavior and TSE), in which mice live for the duration of the experiment while performing the task *ad libitum*, we first explored the capacity of C57BL/6JOlaHsd mice to discriminate frequencies of pure tones, characterizing their just noticeable differences (JNDs). Using the same general task contingencies, we then measured their ability to generalize across frequencies in an auditory latent-inhibition paradigm. One mouse died after anaesthesia and another was removed because it did not drink in the corner during the initial phases of training.

### Fast learning of the discrimination task

Frequency discrimination was tested in a memory-based design. In each trial, mice were presented with one frequency only, and had to decide whether to nose-poke or not. Thus, they could not rely on within-trial same *vs.* different judgements. This design made it possible to compare discrimination with generalization, which was also memory-based and included single-frequency trials. However, this task was also harder than standard frequency discrimination tests, which usually present the two frequencies during the same trial [7]. In consequence, we expected to find rather poor thresholds for tone discrimination.

**Figure 3:**
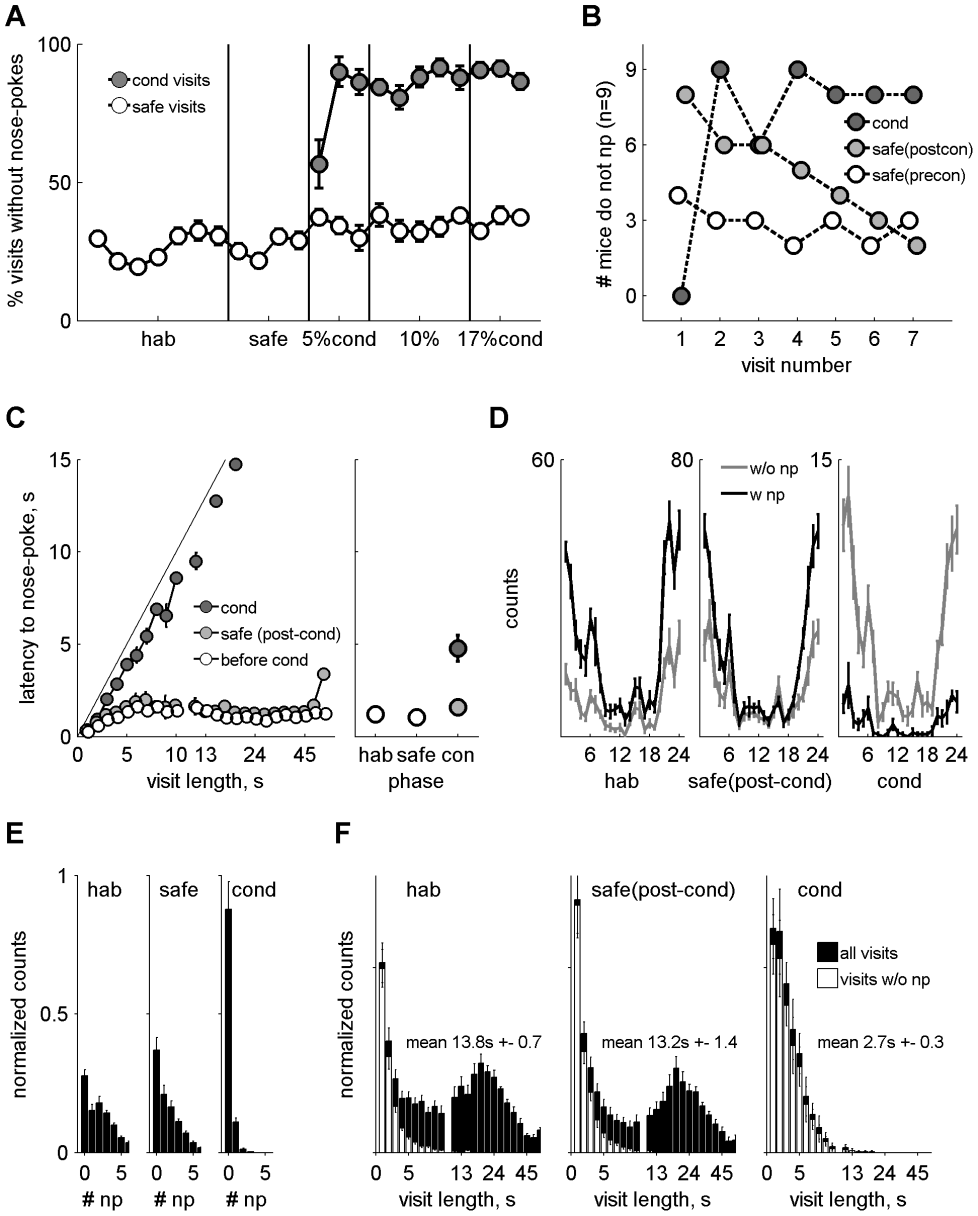
Main discrimination. A. Mean daily performance expressed as the fraction of visits without nose-pokes for the habituation and safe visits (white) and the conditioned visits (grey). B. Single trial performance analysis: # mice that avoided nose-poking (n = 9) during the initial 7 conditioned visits starting with the first visit in which each mouse was punished (dark grey), the 7 safe visits that immediately followed habituation (white), and the initial 7 safe visits after the 1st punished conditioned visit (light grey). C. Left panel: Mean latency to nose-poke as a function of visit length for the safe visits before (white) and during (light grey) conditioning and for the conditioned visits (dark grey). The x axis is linear up to visit length of 10 seconds and logarithmic thereafter. Right panel: mean nose-poke latency per phase and type of visits (colour coding as before). D. Circadian rhythm of visits with (black) and without (grey) nose-pokes in the habituation, safe visits during conditioning and conditioned visits; mean counts. E. Mean distribution of nose-pokes per visits for the different types of visits as in D. F. Mean distribution of visit duration for all visits (black) and visits without nose-pokes (white) for the different types of visits as in D. The x axis is linear up to visit length of 10 seconds and logarithmic thereafter.

Contrary to our expectations, in the main experiment, mice learned to discriminate between the two tones (6670 Hz and 13340 Hz) quickly and reliably, as indicated by the high percentage of conditioned visits without nose-pokes, calculated as the mean value across mice in blocks of 24 hours (Figure 3a). While the mean percentage of safe visits without nose-pokes remained at around 30% throughout, already in the first day of conditioning the percentage of conditioned visits without nose-pokes was around 60%. This increased to 90% by day two (Figure 3a, conditioning phase). There was a significant difference between the level of nose-poking in safe and conditioned visits during the conditioning phase (3-way ANOVA on tone frequency x day x mouse, main effect of frequency F(1,178) = 931.82, p<0.01), and discrimination was borderline significant already on day 1 (F(1,16) = 4.35, p = 0.053).

Mice, in fact, learned the discrimination between their first and second conditioned visits. In their first conditioned visit, all the mice but one (8/9) nose-poked, getting in consequence an air-puff (11% no nose-pokes). Already from the second conditioned visit, however, almost all mice (8/9) refrained from nose-poking. This increase in performance from visit 1 to visit 2 was significant (p<0.02, Fisher's exact test, one-tailed). Figure 3b shows the number of mice that avoided a nose-poke in each of the first 7 conditioned visits, starting with the first visit in which each mouse nose-poked and received an air-puff. Since only 5% of the visits were conditioned initially, mice received an average of 4 conditioned visits on the first day. As a result, the first conditioned visit with a nose-poke had a substantial contribution to the average daily performance of the animals in the first day of conditioning, as seen in Figure 3a. Learning, however, was not quite over with the first conditioned visit, since the behaviour of the mice in the subsequent safe visits was affected as well. In the first few safe visits following the first conditioned visit (Figure 3b, grey circles), mice refrained more than usual from nose-poking. This suggests that mice were more cautious following their first experience of an air puff. However, this cautious behaviour was over within 7 visits, before the second conditioned visit occurred. To illustrate the level of discrimination that was acquired during these few trials, we calculated for each mouse the d' using the hit rate for three conditioned visits that followed the first exposure to an air-puff, and the false alarm rate from the safe visits that immediately followed each of these three conditioned visits. Mean hit or false alarm rates of 0 or 1 were conservatively modified to 0.05 or 0.95, respectively. Six out of the nine mice had a d' above 2 and the mean d' was 1.79±0.40. Thus, unsurprisingly, the high discrimination performance apparent in the average across mice can also be demonstrated in the behavioural performance of the individual mice.

Most mice continued to nose-poke occasionally during conditioned visits (suffering in consequence an air puff), although at a low rate. Their behaviour suggested that they noticed the different frequency: when mice nose-poked during a conditioned visit, the latency to nose-poke was usually longer than the latency to nose-poke in safe visits. Figure 3c shows the mean latency to nose-poke (averaged across mice) for a range of visits lengths separately for the three groups of visits: visits prior to the conditioning phase, safe visits during conditioning, and conditioned visits (also during the conditioning). We performed an Analysis of Covariance (ANCOVA) of the data presented in Figure 3c (latency as a function of group, with visit length as a covariate). There was a main effect of group (F(2,484) = 332, p<0.01) and of visit length (F(1,484) = 74, p<0.01). Most importantly, there was a significant interaction between the two (F(2,484) = 496, p<0.01). Thus, the slopes of the latency as a function of visit length were different in the different groups. Post-hoc comparisons showed that for the conditioned visits, the slope of the latency as a function of visit length was 0.95 (almost 1, as expected, since the latencies were essentially equal to visit length), which was significantly different (p<0.01) from the slopes for the safe visits before conditioning started (0.005) and after conditioning started (0.02); these last two were not different from each other. The interaction was due to the fact that prior to conditioning as well as during safe visits in the conditioning phase, the average latency to nose-poke for any visit length was never larger than 3 seconds (white and light grey points). In the conditioned visits, on the other hand, the latency to nose-poke was essentially the duration of the visit itself. Presumably, in these visits the mouse was uncertain and refrained from nose-poking for a while, then decided to nose-poke nevertheless and terminated the visit immediately following the air puff. Overall, these data indicate that the ability of mice to discriminate between the two frequencies might have been underestimated by the percentage of visits without nose-pokes.

Visits followed a clear circadian rhythm during all phases, with a peak in activity around midnight, as expected from nocturnal animals (see Figure 3d, black line). Activity was low during the light hours and the ratio of visits with nose-pokes decreased slightly during the second half of the light cycle with respect to the other three 6-hour blocks (data not shown). Mice often made more than one nose-poke per visit, usually 1–5, but never more than a dozen (Figure 3e). Visit length showed a bimodal distribution with a sharp peak at 1 second (very short visits) and a broad peak at around 20 seconds (Figure 3f, left and centre panels, black bars).

Visits without nose-pokes constituted about 30% of all safe visits. They were also distributed unevenly throughout the day (Figure 3d, grey line, note the lower count number) with a peak at around midnight. They were generally 1 or 2 seconds in length and never more than 10 seconds (white bars in Figure 3f). Given that a mouse stayed in the corner for more than 3 seconds, the probability of nose-poking was very high. Thus, most of the 30% safe visits with no nose-poking were also the very short visits.

The structure of safe visits was unaffected by the introduction of conditioning visits (Fig. 3d, data shown only for the safe visits that happened during the conditioned phase). There was no significant change in the distribution of nose-pokes in safe visits (3-way ANOVA of probability for N nose-pokes/visit on phase x N x mouse, main effect of phase: F(1,110) = 2.56, p = 0.11; see Figure 3e, middle panel), nor in the distribution of safe visit durations for visits both with (3-way ANOVA of probability for visit duration d on phase x d x mouse, main effect of phase: F(1,399) = 2.11, p = 0.15) and without (F(1,399) = 3.74, p = 0.053; Figure 3f, middle panel) nose-pokes, following the introduction of conditioned visits. There was also no change in the latency to first nose-poke (Figure 3c). The average number of safe visits each day was 61±5 and 67±6 for the safe only and the conditioning phases respectively.

The conditioned visits differed from the safe visits in several aspects. For example, in the rare cases a mouse did nose-poke in one of the conditioned visits, it usually did so only once (Figure 3e, right panel). The bimodal distribution of visit length observed in the safe visits was not present in the conditioned visits (Figure 3f, right panel), with long visits being almost absent from the conditioned visit distribution. There was, in fact, a significant difference between the distribution of durations of visits in the safe and conditioned visits in the conditioning phase (3-way ANOVA of probability of visits duration d on phase x d x mouse, main effect of phase: F(1,399) = 13.99, p<0.01), with the conditioned visits being shorter on average (2.7 s *vs* 13.2 s). Conditioning also had an effect on the latency to nose-poke in the few visits in which there was a nose-poke, as discussed above (Figure 3c). The circadian pattern, on the other hand, was not affected by conditioning (Figure 3d, right panel). A one-way ANOVA on the normalized number of visits across the 24 hour circadian cycle yielded no significant difference between the habituation phase, the safe visits of the conditioning phase and the conditioned visits of the conditioning phase (F(2,69) = 0.03, p = 0.97).

The same analyses, performed for the replications in Göttingen, are reported in the Text S1 and in Figure S1. All the observations reported above were reproduced with these additional groups of mice. The distribution of visit duration was slightly shifted in the Göttingen replications, with the number of short visits peaking at a duration of 3 seconds.

### Just-noticeable differences (JND)

**Figure 4:**
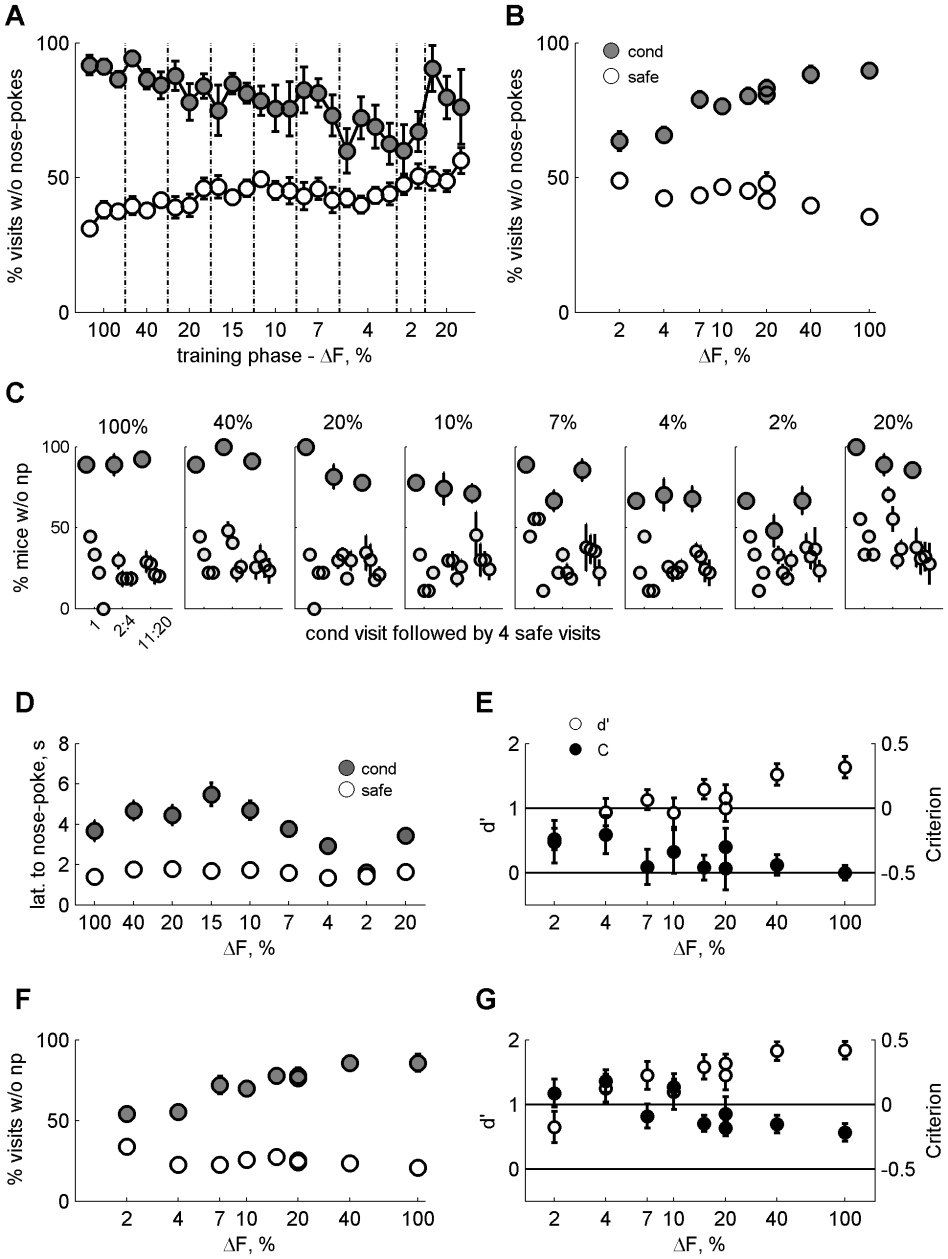
Main JNDs. A. Mean daily performance in fraction of visits without nose-pokes for the safe (white) and conditioned visits (grey) across phase, starting with the last 3 days of ΔF of 100%. The frequencies used were 6670 Hz for the safe tone and 6803, 6937, 7137, 7337, 7670, 8004, 9433, and 13340 Hz for the conditioned tone at ΔFs of 2, 4, 7, 10, 15, 20, 40, and 100% respectively. B. Mean performance as a function of ΔF for safe (white) and conditioned (grey) visits. C. Single trial performance in % mice that avoid nose-poking during the 1st 3 conditioned visits (dark grey), and the 1st 3 safe visits (light grey that follow each of the conditioned visits). D. Mean nose-poke latency as a function of delta F for safe (white) and conditioned (grey) visits. E. Mean d' and C calculation as a function of ΔF. F. The same as in B after removal of visits of 1 second or less duration. G. Mean d' and C as in E but without visits of 1 second or less duration.

In the main experiment, once the mice achieved a stable discrimination performance between tones that were one octave apart, we lowered the conditioned frequency every three days, bringing it progressively closer to the safe tone frequency. The goal was to find the smallest frequency difference at which the animals could perform the task (just-noticeable difference, JND). As the conditioned frequency was lowered (Figure 4a), the mean percentage of conditioned visits without nose-pokes decreased (presumably because the mice decided that the safe tone was presented and nose-poked in consequence), and the mean percentage of safe visits without nose-pokes increased (presumably because the mice decided that the conditioned tone was presented and refrained from nose-poking). A 3-way ANOVA with ΔF x conditioned versus safe visits x mouse resulted in a main effect of ΔF: F(7,344) = 7.9, p<0.01; a main effect of conditioned versus safe visits: F(1,344) = 1.9, p<0.01; and a significant interaction: F(7,344) = 23.48, p<0.01. Some mice could discriminate between frequencies that were as close as 2% apart, and although the difference between safe and conditioned visits without nose-pokes was not very large at that point (63% *vs*. 47%), it was significant (1-way ANOVA on condition x animals, main effect of conditioned versus safe visits: F(1,8) = 11.6, p<0.01). When we moved the conditioned frequency back up to 20% above the safe frequency, performance returned to normal, indicating that the reduction in discrimination performance was not due to a loss of stimulus control.

The development of frequency discrimination in time is studied in Fig. 4c. Each panel corresponds to one ΔF. The performance (as a percentage of mice without nose-pokes) in the first conditioned visit together with the subsequent 4 safe trials is presented first (leftmost dark grey circle), followed by the average performance in conditioned visits 2 to 4 (centre dark grey circle), and in conditioned visits 11 to 20 (rightmost dark grey circle). Each of these is followed by the average performance in the following four safe visits (light grey circles). The leftmost panel, for ΔF of 100%, begins when the probability of appearance of a conditioned tone is already 17%, i.e. 8 days into the conditioning. The performance in this panel represents, therefore, a learned behaviour (in contrast with the data in Fig. 3, which document behaviour at the initial presentations a conditioned tone). On the other hand, the next panel, ΔF of 40%, presents performance from the first presentation of the corresponding conditioned tone. It shows that once the animals knew the task, they immediately avoided nose-poking when confronted with new tones. Presumably, they learned to generalize across tones that were different from the safe tone at 6670 Hz. Simultaneously, they continued to nose-poke in safe visits at approximately the normal rate, even if these visits directly followed a conditioned visit. This pattern held for ΔFs as small as 10% and 7%, breaking down only at ΔFs equal or smaller than 4%.

In conditioned visits in which the animal nose-poked, the mean latency to nose-poke across mice became shorter as the discrimination became more difficult (Figure 4d). In the easiest condition used here, ΔFs of 100%, the latency to nose-poke in conditioned visits was larger than for safe visits. In the most difficult condition (frequency difference of 2%), the average latency to nose-poke was shorter, and not significantly different from the response latency to the safe tone (F(1,16) = 0.87; p = 0.36). This reduction is consistent with the possibility that at least some nose-pokes at very small frequency differences occurred because the conditioned frequency was mistaken for the safe one. Nevertheless, a 2-way ANOVA of latencies (factors: condition (safe *vs*. conditioned) x ΔF) resulted in a significant effect of safe versus conditioned tone (F(1,143) = 40.3, p<0.01), no effect of ΔF (F(8,143) = 1.47, p = 0.17), and no interaction (F(8,143) = 0.55; p = 0.81). It is possible that the large and consistent difference in latency between safe and conditioned tones at all ΔFs except possibly 4% and 2% swamped the effects of ΔF on latency in this analysis.

Correct discrimination between two cues takes place when there is both high avoidance of the conditioned stimulus (hits) and low avoidance of the unconditioned stimulus (low level of false alarms). The d' value (see Methods) takes into account the rates of both hits and false alarms. The threshold for successful discrimination is often considered to be the frequency difference at which d' equals 1. In our case, the average d' (across animals, Figure 4e) measured for the behaviour during the second day of each phase was around 1 for conditioned frequencies with a ΔF of 7%–15% above the safe frequency, and was below 1 for ΔF of 4% and below. This positions the JNDs at 7% (Figure 4e). In fact, almost half the mice (4/9, 44%) had d'>1 at ΔF of 4%. We calculated the threshold for each individual mouse and found that on average individual d' was 1 or above for ΔFs of 7.6%±1.45 (mean ± standard error). This was mainly due to mouse number 3 who had a d' above 1 only for a ΔF of 100%. The other mice had on average d' that was 1 or above for ΔFs of 5.4%±1.25. Figure S2 displays the psychometric functions of all mice.

This way of calculating d', however, underestimated the ability of the mice to discriminate between two tones. The reason is that throughout the experiment, mice did not nose-poke in about 30% of the safe visits. Since most of the visits without nose-pokes were very short (see Figure 3f), we also analysed the data while removing from the analysis any visit that was shorter than 1 second. Short visits represent 18% of all safe visits and 30% of conditioned visits. Most of them did not have nose-pokes (94% and 97%, respectively). They most likely represent visits that were made by the mice in a moment of high activity without an aim to drink. Removing these short visits (Figure 4f) had a large effect on the percentage of safe visit without nose-pokes (compare with Figure 4b), but not so much on the conditioned visits, in which visits without nose-pokes could also have durations above 1 second. In general mean d' values increase when either the number of hits increases or the number of false alarms decreases. In consequence, removing the short visits led to an increase in d' values (Figure 4g), which were clearly larger than 1 for ΔFs of 4% and above. At ΔF of 4%, and 7%, the percentage of mice with d' above 1 was 89% and 78% respectively. Even for ΔF of 2%, 3/9 of the mice (33%) had a d' above 1.We calculated the threshold for each individual mouse and found that on average d' was 1 or above for ΔFs of 4.1%±1.35.

The criterion C (see methods) was also affected by the change in ΔF, independently of whether it was measured with (Figure 4e) or without (Figure 4g) short visits. Either way, the criterion (averaged across mice) became more conservative as ΔF decreased and the decision became harder, increasing overall by about half a standard deviation unit. The increase is expected when the cost of misses (nose-poking in conditioned visits) is higher than the cost of false alarms (not drinking in safe visits). The criterion increased with the removal of short visits, as expected given that the proportion of visits with nose-pokes increased with the removal of the short visits.

Two replications of the discrimination experiment were conducted in Göttingen (See Text S1 and Figure S3). They reproduced all the major results reported here, in spite of small differences in the d' values, which were somewhat lower for each ΔF.

### Generalization

To test generalization across frequencies we used the latent inhibition paradigm in a new group of naïve mice. To elicit latent inhibition, a pre-exposure phase was introduced between the safe-only phase and the conditioned phase. The pre-exposure phase consisted of 83% safe visits (6670 Hz) that did not differ from previous safe visits and 17% pre-exposure visits in which another tone frequency was used as an additional safe frequency that was not associated with any aversive outcome: the mouse could nose-poke and obtain water as in any other safe visit. This frequency varied between 3335 Hz and 15864 Hz in different experimental groups (but was always the same within group); we will use the terms ‘pre-exposure frequency’ and ‘pre-exposure visits’ below to refer to visits in which this additional frequency was presented. Subsequent to the pre-exposure phase, all mice were conditioned at 13340 Hz, irrespective of their pre-exposure frequency.

**Figure 5:**
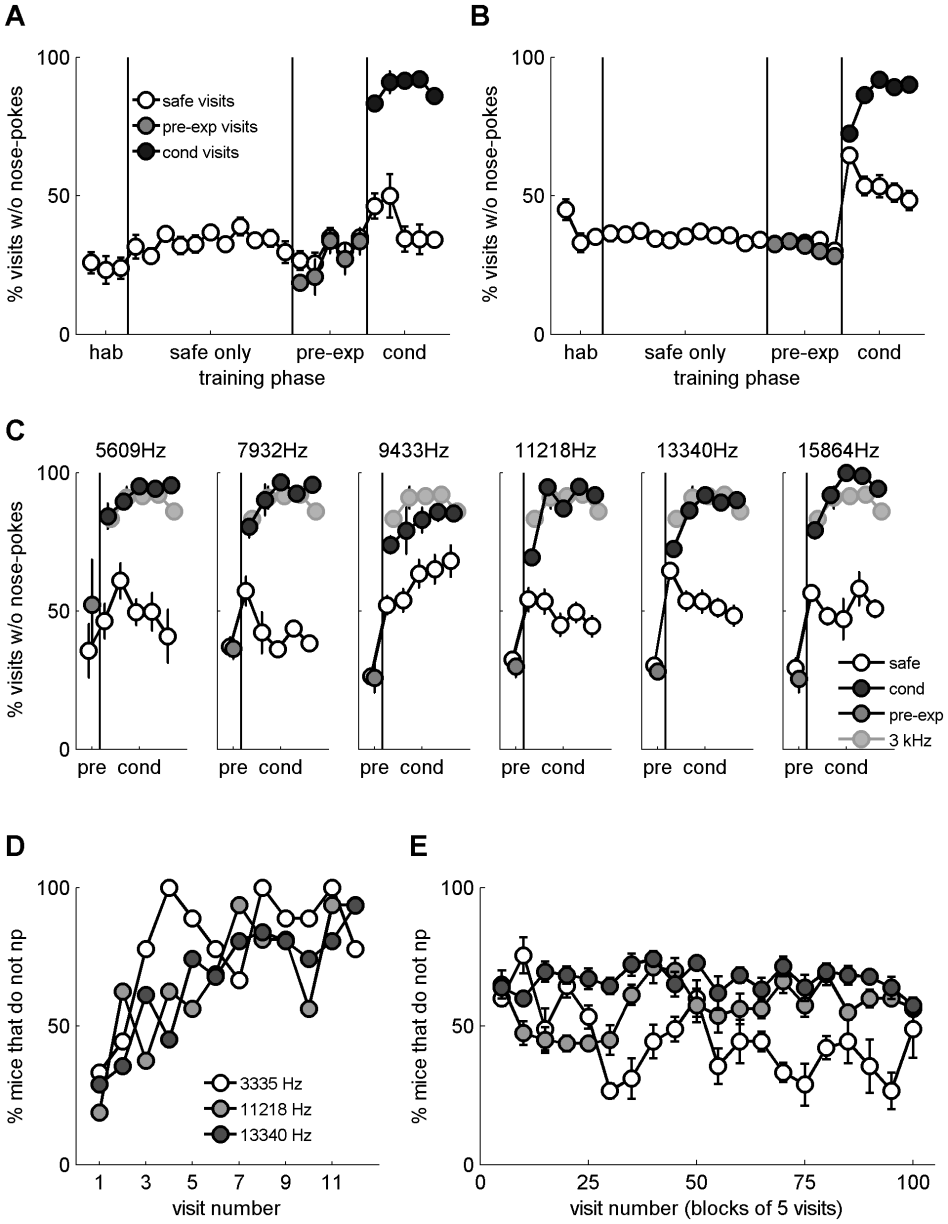
Latent inhibition. A. Mean daily performance (% visits without nose-pokes) of mice pre-exposed to 3335 Hz. White dots are habituation or safe visits and grey dots are the remaining 17% of visits (pre-exposure or conditioned visits). B. As in A for mice pre-exposed to 13340 Hz. C. As in A but showing only the last day of the pre-exposure phase and 5 days of conditioning for all the pre-exposure frequencies used, except for the groups pre-exposed to 3335 Hz, whose performance during conditioning appears in all plots as light grey circles. D. Performance in the single visits for the 1^st^ 12 conditioned visits after pre-exposure to 3335 Hz (white), 11218 Hz (light grey), or 13340 Hz (dark grey). E. The same as in D for the 1^st^ 100 safe visits, in blocks of 5 trials, that followed the first conditioned visits.

The introduction of the pre-exposure frequency alone had no effect on the behaviour (Figure 5a for 3335 Hz, 5b for 13340 Hz) and mice continued to avoid nose-poking on average in about 30–40% of both the safe and the pre-exposure visits. This was expected given that the pre-exposure visits were not behaviourally different from the safe visits.

When mice that have been pre-exposed to 3335 Hz were conditioned to 13340 Hz (Figure 5a), they learned to avoid nose-poking in conditioned visits already on day 1, with an average of 83% of conditioned visits without a nose-poke. There was also a small and temporary increase in the mean rate of safe visits without nose-pokes, suggesting that the mice became more conservative in their decision to nose-poke.

In contrast, pre-exposing mice to the 13340 Hz tone elicited latent inhibition of the conditioned response once conditioning started (Figure 5b), with only 70% of conditioned visits (averaged across mice) without nose-pokes over the first 24 hours. The effect was more pronounced in the first 6 hours; these fast changes in behaviour during the first day will be analysed below. By day 2 of conditioning, the mice pre-exposed to 13340 Hz reached a performance level of 90% (averaged across mice). In addition to the latent inhibition, there was a noticeable effect on the performance in the safe visits. After the beginning of conditioning, these mice, like those pre-exposed to 3335 Hz, increased the number of safe visits without nose-pokes. This generalization of the conditioning to the safe tone was more long lasting than the latent inhibition, and suggests the use of a more conservative criterion in the decision to nose-poke in this group, even when exposed to the safe tone.

Frequencies other than 13340 Hz also elicited latent inhibition. Figure 5c shows the average rate of visits without nose pokes in the last day of pre-exposure and for 5 days of conditioning for all pre-exposure frequencies, superimposed on the same data for the group pre-exposed to 3335 Hz (light grey circles). Pre-exposure frequencies close to 13340 Hz elicited latent inhibition – for example, the rate of conditioned visits without nose pokes in the first day of conditioning was essentially the same for pre-exposure frequency of 11218 Hz as for pre-exposure frequency of 13340 Hz. Furthermore, the generalization of the conditioning to the safe tone was also apparent for frequencies close to 13340 Hz. This increase was longer lasting than the latent inhibition itself.

We analysed in detail the way learning progressed through the first day of conditioning in three groups: mice pre-exposed to 3335 Hz (n = 9), to 11218 Hz (n = 16), and to 13340 Hz (n = 31; Figure 5d). The percentage of mice that nose-poked in each of the first 10 conditioned visits was analysed using a 2-way ANOVA (factors: pre-exposure frequency x visit number). We chose 10 trials because this is the least number of trials at which one group achieved reasonable performance in the conditioned visits (at least 75% of the mice avoided nose-poking in 3 consecutive visits). There was a significant effect of group (F(2,18) = 3.7; p = 0.046) and a significant effect of visit number (F(9,18) = 5.6; p<0.01). To identify the cause for the significant group effect, a post-hoc paired t-test on the percent of mice that nose-poked in each of the first 10 trials of the groups pre-exposed to 3 kHz and to 13 kHz resulted in a significant difference (t(9) = 2.49; p = 0.03). The first day of conditioning had a group mean of 106, 104.5 and 124.6 visits for each group respectively. Of these, an average of 19.2, 16.1 and 20.3, respectively, were conditioned. Thus, a small difference in the number of conditioned visits without nose-pokes had a strong impact on the overall performance.

We showed above that in the simple conditioning task (without pre-exposure) mice learned the discrimination with a single exposure to the conditioning frequency. There are two reasons that might explain why in the generalization task learning was slower, even in the group pre-exposed to 3335 Hz. In the simple conditioning task used for measuring frequency discrimination, the conditioned tone was first presented in only 5% of visits, and not 17% as in the present task. We know (data not shown) that increasing this probability leads to a decrease in the number of hits as well as an increase in the number of false alarms, which might be the reason why in the current task the best group needed about 4 trials to learn the discrimination. Alternatively, since the mice have already been exposed to two safe tones they might generalize some of that learning to any new frequency, in which case the slower learning is really a component of the latent inhibition which is common to all pre-exposure frequencies.

Figure 5e shows a trial-by-trial analysis of the generalization of conditioning to the safe frequency, conducted on the average performance across mice. In the initial safe visits of this phase, the increase in the percentage of mice that did not nose-poke was apparent for all pre-exposure frequencies, but it was longer lasting in the group pre-exposed to 13340 Hz, in which this form of generalization lasted several days (see Figure 5b). Even when testing as many as the first 100 safe visit after conditioning began, an ANOVA on the percentage of animals that nose-poked in each visit across the three behavioural groups (group x sequential visit number) revealed an effect of group (F(2,198) = 59.9; p<0.01) but no effect of sequential visit number (F(99,198) = 0.73; p = 0.96). A post-hoc two sample t-test comparing the group pre-exposed to 3 kHz and that pre-exposed to 13 kHz revealed a significant effect of group (t(99) = −10.8; p<0.01).

**Figure 6:**
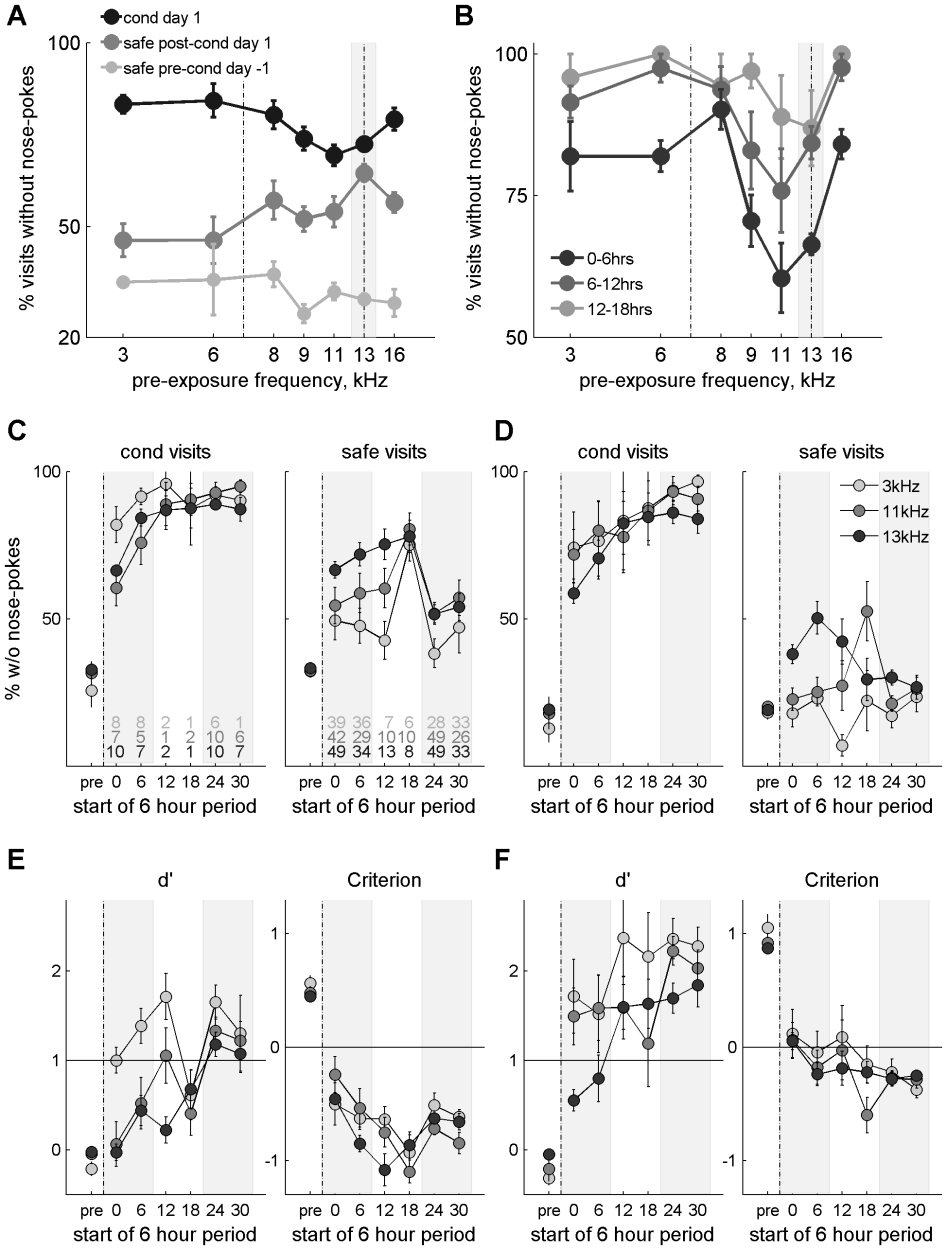
Behavioural tuning curve of latent inhibition. A. Mean performance (% visits without nose-pokes) on the first day of conditioning across pre-exposure frequencies for the conditioned visits (dark grey) and safe visits (mid grey). Small light grey dots represent the performance on the safe visits on the last day of the pre-exposure phase, for comparison. B. The same as in A for the conditioned visits only, in blocks of 6 hours (color coded) for the first 18 hours after the start of conditioning. C. Mean percentage visit without nose-pokes for conditioned (left) and safe (right) visits, in blocks of 6 hours for mice pre-exposed to 3335, 11218 or 13340 Hz. The lines with numbers are the number of conditioned (left) and safe (right) visits per time block for each of the three groups (color-coded as for dots) D. The same as in C, but removing from the analysis visits that were 1 second or less in duration. E. Mean d' values (left) and C (right) calculated using the same conditions as in C. F. As E but removing visits that were 1 second or less in duration.

Latent inhibition was therefore quantified by the rate of visits without nose-pokes during the first day of conditioning. The frequency tuning curve of latent inhibition is displayed in Figure 6a. From day 1 of conditioning, all animals faced the same behavioural contingencies: 13340 Hz was used as the conditioned frequency and was presented in 17% of the visits, while 6670 Hz tones were used in the remaining, safe, visits. Differences between the groups are, therefore, due to the differences in the frequencies used during the pre-exposure stage, before conditioning started. A 2-way ANOVA on the fraction of visits without nose-pokes (factors: pre-exposure frequency and visit type) showed no main effects of pre-exposure frequency (F(6,164) = 1.78; p = 0.10), but a significant main effect of visit type (F(1,164) = 128.86; p<0.01). Most importantly, there was a highly significant interaction between pre-exposure frequency and visit type (F(6,164) = 5.4; p<0.01). Focusing on the conditioned visits only, a one-way ANOVA revealed a significant effect of pre-exposure frequency on the percentage of visits without nose-pokes (F(6,82) = 3.87; p<0.01). Post-hoc comparisons (Tukey's honestly significant difference) showed that frequencies between 7932 Hz and 15864 Hz elicited significant latent inhibition of the conditioning to the 13340 Hz tone, while frequencies outside this range did not.

Latent inhibition was transient: the effect was very pronounced during the 6 hours following the first conditioned visit (Figure 6b, black) but had practically disappeared after 12 hours (light grey). Interestingly, it was slightly more pronounced in the group pre-exposed to 11218 Hz than in the group pre-exposed to 13340 Hz itself, even though the generalization to the safe tone was stronger for a pre-exposure frequency of 13340 Hz. We performed an Analysis of Covariance on the conditioned visits without nose-pokes as a function of pre-exposure frequency (Figure 6b), with time (consecutive 6 hour periods) as a covariate. There was a significant effect of time (F(1,203) = 45, p<0.01), due to the progress of the learning process. The rate of visits without nose pokes decreased on average by about 10% for each 6 hour blocks. Importantly, there was a main effect of pre-exposure frequency (F(6,203) = 4, p<0.01) on the percentage of visits without nose-pokes. No significant interaction was found. Post-hoc comparisons showed that the percentage of visits without nose-pokes was lower at 11.2 and 13.4 kHz than at the other frequencies. Thus, during the initial 6 hours of conditioning, latent inhibition represented a reduction in mean performance of 19.5% for the group pre-exposed and conditioned to 13 kHz and of 26.8% for the group pre-exposed to 11 kHz relative to the group pre-exposed to 3.3 kHz. By the 18^th^ hour after the beginning of conditioning the performance was reduced only by 12.5% and 7.3%, in the two groups respectively relative to the group pre-exposed to 3.3 kHz.

The effect that conditioning had on the safe visits (generalization to the safe tone; Figure 6a, dark grey dots) also depended significantly on the pre-exposure frequency (one-way ANOVA on pre-exposure frequency, F(6,82) = 3.46; p<0.01), although it could be observed to a certain extent at all pre-exposure frequencies used (compare dark and light grey circles in Figure 6a). As discussed above, it was substantially longer-lasting than the latent inhibition itself.

We performed signal detection analysis on the behaviour of the groups pre-exposed to 3335 Hz, 11218 Hz and 13340 Hz. Animals pre-exposed to 3335 Hz had no LI and the least generalization to the safe tone, animals pre-exposed to 11218 Hz had strong LI and mild generalization to the safe tone, and animals pre-exposed to 13340 Hz had LI and strong generalization to the safe tone. We measured d' and C (see Methods) with and without short visits, similarly to the analysis for the frequency discrimination experiment. Figures 6c and d show the percentage of conditioned (left) and safe (right) visits without nose-pokes in 6 hour intervals from the beginning of conditioning. These are compared with the average behavioural performance during the pre-exposure phase (pre; for the conditioned visits we used the pre-exposure visits which were not conditioned and should not behave differently from the safe visits during that phase). The first time points on these figures display data already shown in Figures 5d and 5e, except that here the time resolution is coarser and the data covers a longer period of time.

Both the LI and the generalization to the safe tone are readily apparent in Figure 6c (as in Figure 5d). LI is evident in the longer time it took the groups pre-exposed to 11218 and 13340 Hz to reach the level of conditioning (nose-poke avoidance) of the group pre-exposed to 3335 Hz. Only after 12 hours of conditioning did all the groups reach the same performance level for the conditioned tone. The generalization to the safe tone was larger in the group pre-exposed to 13 kHz and long lasting when present (as in Figure 5e). Since conditioning begun with the dark phase, the block starting at 18 hours after start of conditioning coincide with the light phase, and, therefore, with low activity (see number of trials for each period in Figure 6c). This pattern of lower nose poking during the second half of the light cycle was also observed in the mice that underwent discrimination training, as described above. Removing the short visits from the analysis (Figure 6d) had a strong effect on the safe visits (right panel) with the generalization to the safe tone disappearing in all but the group pre-exposed to 13 kHz.

We derived d' and C from these data. In animals pre-exposed to 3335 Hz, the average d' was close to threshold value of 1 already during the first 6 hours of conditioning and improved fast during the following 18 hours (Figure 6e, left). Discrimination, measured through d' values, was worse for pre-exposure frequencies closer to the conditioned frequency, as expected, reaching a minimum in mice pre-exposed to 13340 Hz. These mice showed a mean d' of essentially 0 during the first 6 hours of conditioning, reflecting their identical behaviour in the presence of either conditioned or safe tones. The mice reached d'>1 only during the 2^nd^ day of conditioning, when the generalization to the safe tone had partially subsided. Removing visits shorter than 1 second (Figure 6f, left), had, as expected, little effect on the values measured over the pre-exposure phase but increased the values of d' during conditioning, because the main effect of removing the short visits was a decrease in the number of safe (or pre-exposure) visits without nose-pokes. There was still a distinct delay in the increase of d' in the mice exposed to 13340 Hz.

The criterion C (see Methods) showed the largest change following the transition from pre-exposure to conditioning, and a more gradual change thereafter (Figure 6e). During the pre-exposure phase values of C were high because the number of nose-pokes was high for both pre-exposure and safe visits. Immediately after the start of conditioning, the number of nose-pokes decreased in both conditioned and safe visits, leading to a lower criterion that reflected this more conservative behaviour. Removing the short visits from the analysis (Figure 6f) led to an overall increase in C values during conditioning because of the increase in the proportion of safe visits with nose-pokes, but did not affect the overall pattern of change. C remains largely negative (conservative), as expected from a higher cost for nose-pokes in conditioned visits than from failures to nose poke in safe visits.

## Discussion

Using a novel behavioural paradigm, we measured both discrimination and generalization for pure tone frequency in C57BL/6J mice. Discrimination thresholds (d' = 1) were 4%-7%, although mice could discriminate significantly above chance frequencies as close as 2% apart. In spite of these small discrimination thresholds, mice generalized spontaneously over a range of at least 0.5 octave. Thus, mice generalize over frequencies that are easily discriminable.

The Audiobox allows training and testing in the animal living quarters, where animals live in groups and handling is minimized to the weekly cleaning. The mice were neither food- nor water-deprived: performing the task ensured their access to water. This setting makes it possible for each mouse to develop a spontaneous pattern of behaviour. We saw, for example, very clear circadian rhythms with their activity, peaking around midnight. Mice made numerous visits to the corner but did not nose-poke in about one third of the safe visits. This behaviour presumably reflects their natural curiosity and may be characteristic of each strain or genetic background. It most probably does not reflect lack of motivation: the visits without nose-pokes were short and often occurred after a period of inactivity but sometimes also occurred between visits with nose-pokes. These short visits are presumably a feature of tasks in which performance is *ad libitum*. Since they represent a problem for estimating the true level of false alarm rates in these tasks, future strategies could be devised to reduce these visits for studies that require low levels of false alarms.

C57BL\6J mice carry a mutation in the Ahr1 gene that leads to age-related hearing loss [13]. By about 3 months of age hearing thresholds start to deteriorate, initially only for high frequencies but with time for progressively lower frequencies [14]–[15]. For the ages (<14 weeks) and frequencies (<16 kHz) used in this study, we did not expect any relevant hearing loss [14], [16]–[17]. The oldest mice used here finished their training when they were 14 weeks old but reached levels of discrimination comparable to those of the young mice. Thus, age-related hearing loss did not affect the conclusions of this study.

Mice learned to discriminate between two frequencies that were one octave apart between their first and second conditioned visits. They achieved discrimination thresholds that were comparable or only slightly larger than other thresholds obtained in rodents. In highly-trained mice, frequency discrimination thresholds are at 3% for frequencies around 8 kHz [18], [8], and at 2.4% in young mice for frequencies around 16 kHz [19]. In rats, values vary between 6% [20] and 3% [3]. Typically, in these studies different tone frequencies were presented within the same trial and animals responded to the change in tone frequency. In contrast, our paradigm did not allow the mice to compare between the safe and the conditioned tone within the same trial. The thresholds we measured required comparison with a memory trace of the safe tone, and therefore presumably represent the acuity of an internal representation of the frequency axis. Nevertheless, perceptual acuity was only slightly affected.

Mice learned to discriminate frequencies very quickly by comparison with other operant auditory protocols: significant frequency discrimination was observed already on the first day of conditioning. Admittedly, conditioning occurred after 7 days in the Audiobox. In contrast, NMRI mice trained with a go/no-go paradigm required about 600 trials to reach asymptotic performance [21], and CBA/CaJ mice may need several weeks to learn a go/no-go tone/no-tone discrimination [22]. It is plausible that the continuous and *ad-libitum* training in the home cage is at least partially responsible for the fast learning we observed. Indeed, it has been shown previously that differences in housing conditions and training paradigms can lead to differences in auditory thresholds [11], [23]–[24], [2] for review.

Mice spontaneously categorized all new tone frequencies as dangerous once they learned the paradigm. This is reflected in Figures 5b, 6b and 6d: mice tended to avoid nose-pokes to new frequencies from their first presentation. This category had surprisingly steep boundaries, as reflected in the good frequency JND we report.

Despite their relatively small JND, mice generalized across a wide frequency band (half an octave) of pre-exposure frequencies, as judged by the level of latent inhibition in the response to 13 kHz induced by the pre-exposure to frequencies ranging between 3 and 16 kHz for different groups of mice. A similar generalization has been shown before in the rabbit using the eyelid classical conditioning, and tones as predictive stimuli [25]–[26], although animals in these studies received more pre-exposure trials than our mice (>1000 and 450 presentations, respectively). Lengthening the pre-exposure period leads to an increase in the level of latent-inhibition in rats [27].

The behavioural tuning curve of latent inhibition was asymmetrical, narrower for frequencies above the conditioned tone. Although the asymmetrical shape, as well as the precise half-width of the behavioural tuning curve of latent inhibition, could result from the presence and position of the safe tone 1 octave below the conditioned tone, this is unlikely. Indeed, the generalization tuning-curves in the studies of Siegel [25] and Solomon and Moore [26] were also asymmetrical around the conditioned tone (on a logarithmic scale) and steeper towards the higher frequencies, although no safe tone was used. In these studies the width of the behavioural tuning curve for LI generalization was at least 1 octave in the Siegel study [25], which uses frequencies between 0.5 and 4 kHz with 1 kHz resolution; and at least half octave in the Solomon and Moore study [26], using pure tones between 4 and 10 kHz with a resolution of 4 kHz. These widths are comparable to ours.

Tuning curve widths of half an octave are not uncommon in C57Bl/6 mouse nerve fibres for similar frequency ranges and for lower intensities than those used here [28]–[30]. Similarly, psychoacoustical measurements in mice suggest rather wide peripheral channels [31], which may span over half an octave [32]. It is commonly accepted that the bandwidth of the peripheral channels roughly corresponds to that of auditory nerve fibres [33], although the properties of the peripheral channels are determined only at the level of the inferior colliculus [34]. The small frequency differences that can be discriminated by mice are therefore an example of hyperacuity, which is usually associated with integration of information across multiple peripheral sensors, although it could potentially be achieved by using the steep high-frequency slopes of the tuning curves of selected auditory nerve fibers [29]; see [35]. On the other hand, the shape of the behavioural tuning curve of the latent inhibition is suggestively similar to the stylized tuning curve of auditory nerve fibres and to responses in inferior colliculus, where neurons with wide and asymmetrical tuning curves have been recorded [36], and where perceptual categorization has been suggested to occur [34], [37]. Thus, our results may suggest that generalization is determined by the tuning width of the peripheral filters, either through categorization processes in the inferior colliculus, or through an internal comparison between the level of activation of the auditory midbrain to the conditioning and the pre-exposed frequency. Such an account would require the existence of a memory trace of the excitation pattern evoked by the pre-exposure frequency, presumably stored in higher brain areas such as auditory cortex. These speculations may be directly tested by recording neuronal responses in the auditory midbrain.

In addition to the latent inhibition, we also observed an increase in the number of ‘false alarms’ - safe visits without nose-pokes. This increase was particularly prominent in the group pre-exposed to the conditioned frequency itself. This generalization of the ‘cautiousness’ to the safe tone was, unlike latent-inhibition itself, long lasting, and was due to reduced discriminability (d') between the two tones, rather than a more conservative threshold for nose poking (C). Thus, the generalization to the safe tone could reflect, in part, the stress levels of the animals, who learned to avoid the conditioned tone but were left with the impression that apparently safe stimuli might turn nasty without warning: ‘He who was scalded with boiling water is cautious with cold’.

We show that mice can be trained to perform auditory discriminations of different complexity quickly, reliably, and with minimum handling. This might prove useful in behaviourally characterizing the growing number of mice with auditory system-related mutations [38]. Most importantly, the difference in the width of the behavioural tuning curve due by task design opens a door to study the link between perception and cognition.

## Supporting Information

Figure S1 **Discrimination (Göttingen replications).** A–D is for the mice that begun with 9 weeks of age. A. Mean daily performance expressed as the fraction of visits without nose-pokes for the habituation and safe visits (white and light grey) and the conditioned visits (black and dark grey) for the group that had 6670 Hz as safe and the group that had 13340 Hz as safe. B. Single trial performance analysis across all mice: # mice that avoid nose-poking (n = 18) during the 1st 7 conditioned visits starting with the first visit in which each mouse received an air-puff because it nose-poked (dark grey), the very 1st 7 safe visits after habituation (white), and the 1st 7 safe visits after the 1st punished conditioned visit light grey). C. Mean distribution of nose-pokes per visits in the habituation, safe visits during conditioning and conditioned visits. D. Mean distribution of visit duration for all visits (black) and visits without nose-pokes (white) in the habituation, safe visits during conditioning and conditioned visits. The x axis is linear up to visit length of 10 seconds and logarithmic thereafter. E–F is for the mice that begun with 5–6 weeks of age. E. Mean daily performance expressed as the fraction of visits without nose-pokes for the habituation and safe visits (white and light grey) and the conditioned visits (black and dark grey). B. Single trial performance analysis across all mice: # mice that avoid nose-poking (n = 6) during the 1st 7 conditioned visits starting with the first visit in which each mouse received an air-puff because it nose-poked (dark grey), the very 1st 7 safe visits after habituation (white), and the 1st 7 safe visits after the 1st punished conditioned visit light grey).
(TIF)

Figure S2 **Individual JNDs (Jerusalem replications).** A. Individual psychometric curves for the 9 mice used in Jerusalem as a measure of d' values for each ΔF used. B. The mean psychometric curve (dark) is plotted over a background of individual psychometric curves (light gray). The horizontal and vertical dotted lines represent the level for a d' of 1 and a ΔF of 10%, respectively.
(TIF)

Figure S3 **JNDs (Göttingen replications).** A–C is for mice that begun with 9 weeks of age and D–F for mice that begun with 5–6 weeks of age. A and D. Mean daily performance in fraction of visits without nose-pokes for the safe (white) and conditioned visits (grey) across phase, starting with the last 3 days of ΔF of 100%. B and E. mean d' as a function of ΔF with (grey) and without (white) removal of visits of 3 seconds or less duration. C and F. percentage mice that avoid nose-poking in the first 20 conditioned visits and subsequent safe visits in each of the phases, where the 100% phase begins with the lasts 3 days of conditioning at ΔF of 100%. The first dark grey data point is the first conditioned trial in that phase, the second grey point is the mean performance in conditioned trials 2 to 10, and the third grey point is the mean performance in trials 11 to 20. The light grey points are the 4 safe visits that follow each conditioned trial.
(TIF)

Table S1 **Sequence of conditioned frequencies used for JND assessment in the Göttingen replications.**
(DOCX)

Text S1 **Description of Göttingen discrimination and JND replications.**
(DOCX)
