# Supplementary figures and images for: Frequency Tuning in the Behaving Mouse: Different Bandwidths for Discrimination and Generalization
Source: PLoS One. 2014 Mar 14;9(3):e91676. doi: 10.1371/journal.pone.0091676 (PMC3954732; doi:10.1371/journal.pone.0091676)

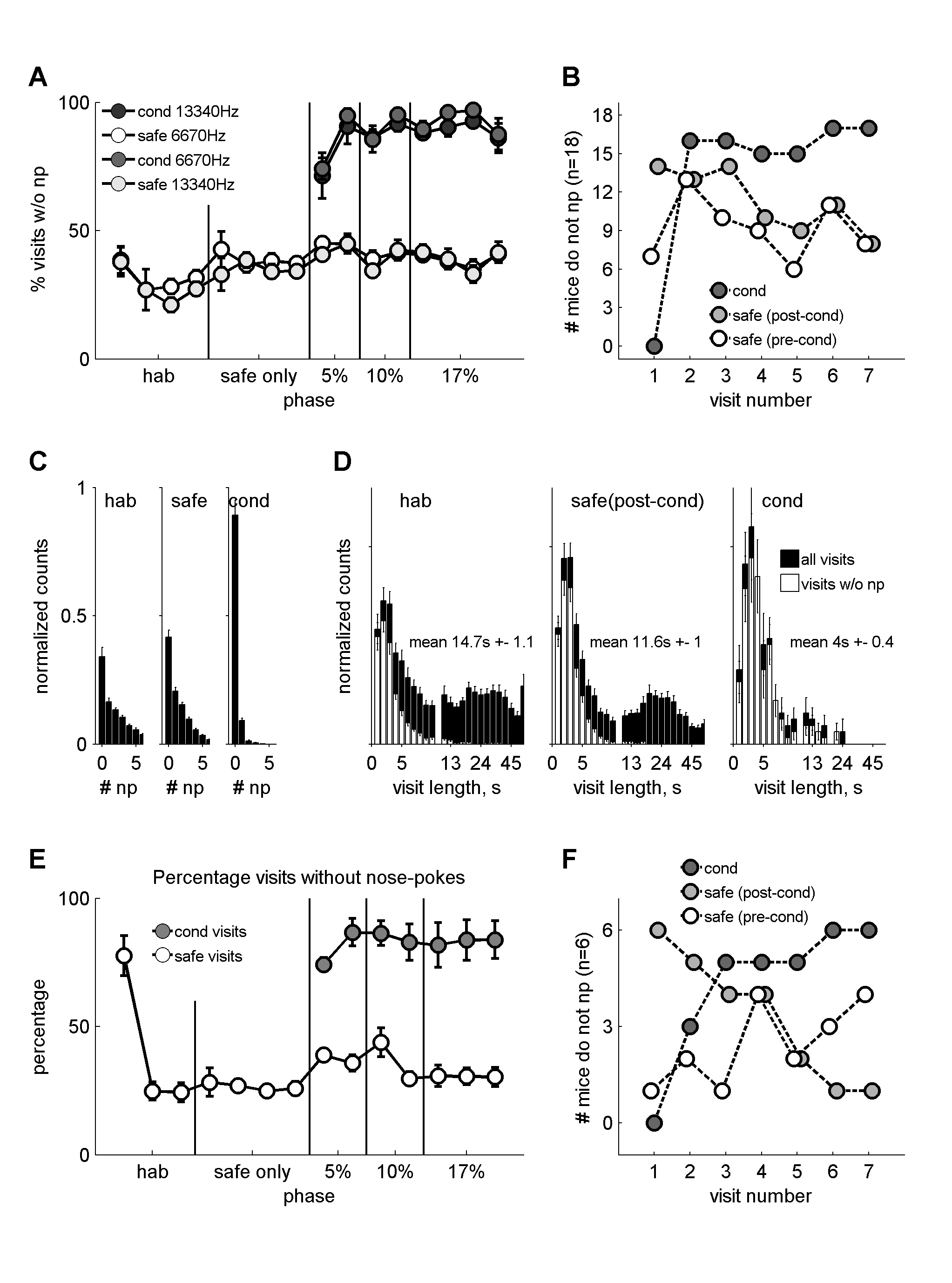

Supplement: Figure S1 — Discrimination (Göttingen replications). A–D is for the mice that begun with 9 weeks of age. A. Mean daily performance expressed as the fraction of visits without nose-pokes for the habituation and safe visits (white and light grey) and the conditioned visits (black and dark grey) for the group that had 6670 Hz as safe and the group that had 13340 Hz as safe. B. Single trial performance analysis across all mice: # mice that avoid nose-poking (n = 18) during the 1st 7 conditioned visits starting with the first visit in which each mouse received an air-puff because it nose-poked (dark grey), the very 1st 7 safe visits after habituation (white), and the 1st 7 safe visits after the 1st punished conditioned visit light grey). C. Mean distribution of nose-pokes per visits in the habituation, safe visits during conditioning and conditioned visits. D. Mean distribution of visit duration for all visits (black) and visits without nose-pokes (white) in the habituation, safe visits during conditioning and conditioned visits. The x axis is linear up to visit length of 10 seconds and logarithmic thereafter. E–F is for the mice that begun with 5–6 weeks of age. E. Mean daily performance expressed as the fraction of visits without nose-pokes for the habituation and safe visits (white and light grey) and the conditioned visits (black and dark grey). B. Single trial performance analysis across all mice: # mice that avoid nose-poking (n = 6) during the 1st 7 conditioned visits starting with the first visit in which each mouse received an air-puff because it nose-poked (dark grey), the very 1st 7 safe visits after habituation (white), and the 1st 7 safe visits after the 1st punished conditioned visit light grey). (TIF) [file pone.0091676.s001.tif]

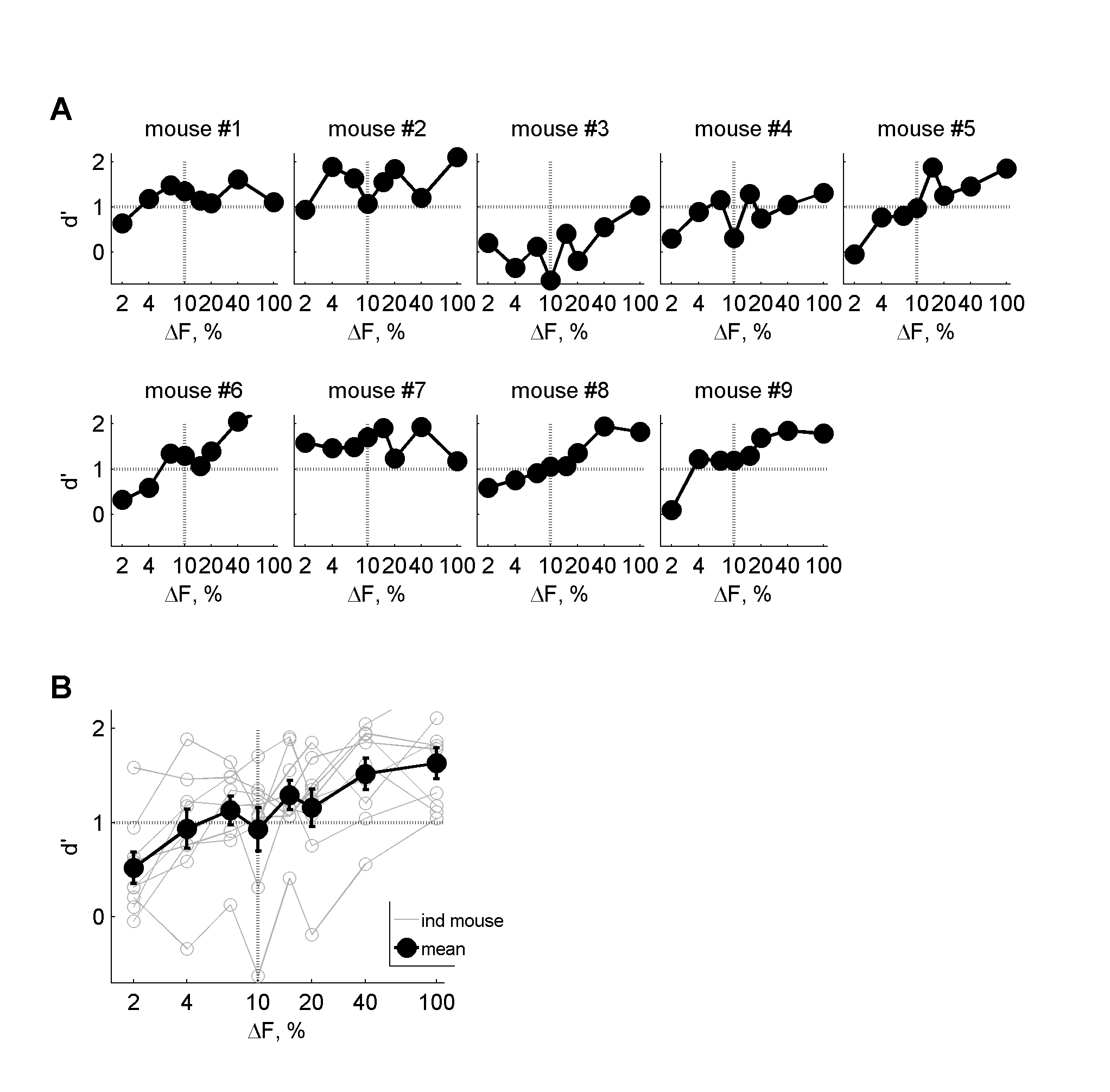

Supplement: Figure S2 — Individual JNDs (Jerusalem replications). A. Individual psychometric curves for the 9 mice used in Jerusalem as a measure of d' values for each ΔF used. B. The mean psychometric curve (dark) is plotted over a background of individual psychometric curves (light gray). The horizontal and vertical dotted lines represent the level for a d' of 1 and a ΔF of 10%, respectively. (TIF) [file pone.0091676.s002.tif]

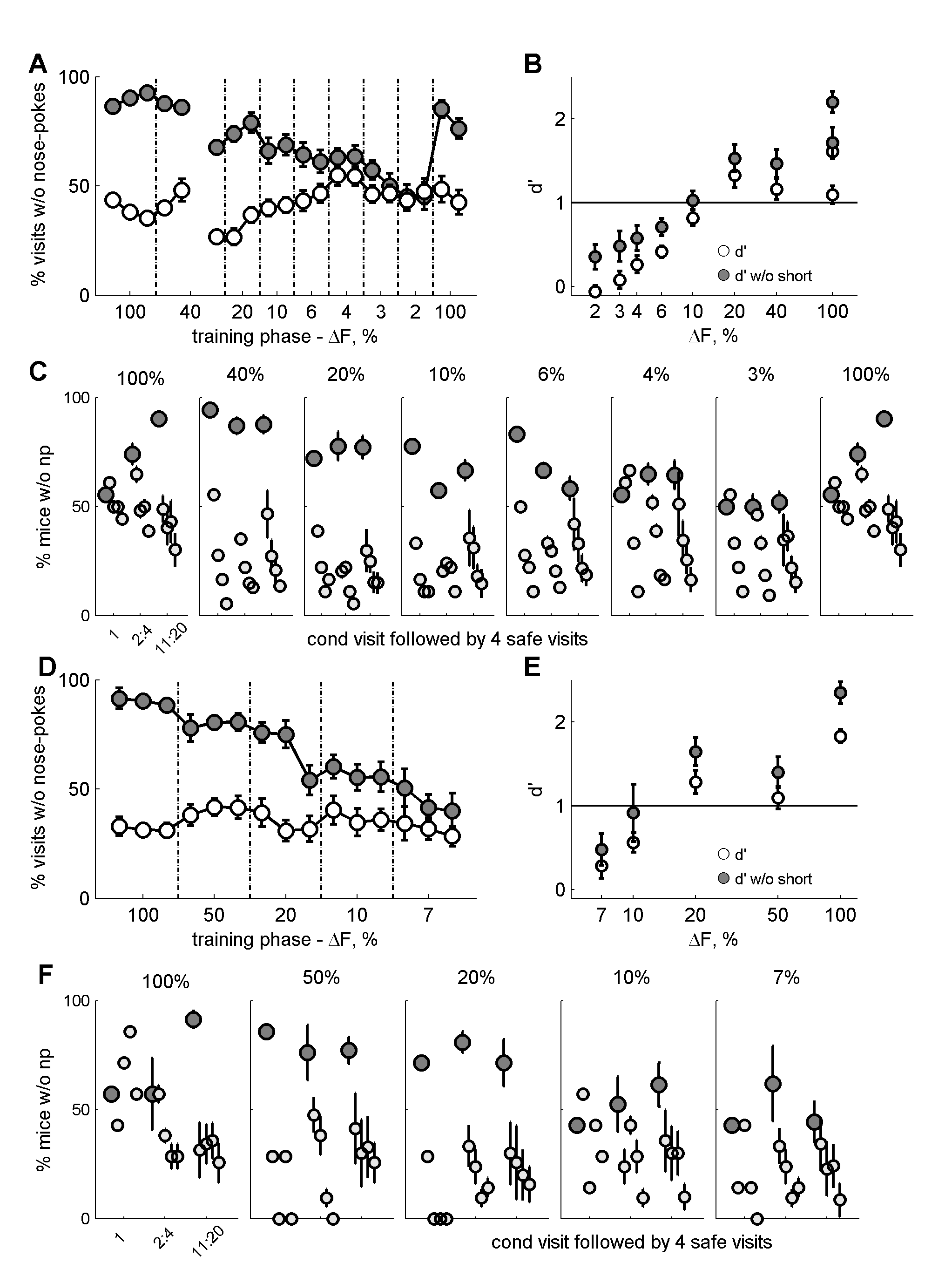

Supplement: Figure S3 — JNDs (Göttingen replications). A–C is for mice that begun with 9 weeks of age and D–F for mice that begun with 5–6 weeks of age. A and D. Mean daily performance in fraction of visits without nose-pokes for the safe (white) and conditioned visits (grey) across phase, starting with the last 3 days of ΔF of 100%. B and E. mean d' as a function of ΔF with (grey) and without (white) removal of visits of 3 seconds or less duration. C and F. percentage mice that avoid nose-poking in the first 20 conditioned visits and subsequent safe visits in each of the phases, where the 100% phase begins with the lasts 3 days of conditioning at ΔF of 100%. The first dark grey data point is the first conditioned trial in that phase, the second grey point is the mean performance in conditioned trials 2 to 10, and the third grey point is the mean performance in trials 11 to 20. The light grey points are the 4 safe visits that follow each conditioned trial. (TIF) [file pone.0091676.s003.tif]
