# Supplementary material for: Frequency Tuning in the Behaving Mouse: Different Bandwidths for Discrimination and Generalization
Source: PLoS One. 2014 Mar 14;9(3):e91676. doi: 10.1371/journal.pone.0091676 (PMC3954732; doi:10.1371/journal.pone.0091676)
Supplement: Table S1 — Sequence of conditioned frequencies used for JND assessment in the Göttingen replications. (DOCX) [file pone.0091676.s004.docx]

**Table S1**

|  | Conditioned tone (% w respect to safe, Hz) | | | | | | | | |
| --- | --- | --- | --- | --- | --- | --- | --- | --- | --- |
| Safe tone (Hz) | ∆f 100% | ∆f 40% | ∆f 20% | ∆f 10% | ∆f 7% | ∆f 6% | ∆f 4% | ∆f 3% | ∆f 2% |
| 6670 | 13340 | 9433 | 8004 | 7337 |  | 7070 | 6937 | 6870 | 6803 |
| 13340 | 6670 | 9529 | 11117 | 12127 |  | 12585 | 12827 | 12951 | 13078 |
| 7000 | 14000 | 9900 | 8400 | 7700 | 7490 |  |  |  |  |

*Table S1. Sequence of conditioned frequencies used in the discrimination experiments*
