# Supplementary material for: Frequency Tuning in the Behaving Mouse: Different Bandwidths for Discrimination and Generalization
Source: PLoS One. 2014 Mar 14;9(3):e91676. doi: 10.1371/journal.pone.0091676 (PMC3954732; doi:10.1371/journal.pone.0091676)
Supplement: Text S1 — Description of Göttingen discrimination and JND replications. (DOCX) [file pone.0091676.s005.docx]

**Text S1**

**Discrimination - Göttingen experiments**

A subset of experiments was replicated in Göttingen to ensure reproducibility of the pattern of results obtained with this new paradigm.

In total we ran 3 replications of the discrimination task. The first one was run in Jerusalem and is described in the main text. The second experiment was run in Göttingen and used 18 mice (Harlen, Germany; C57BL/6JOlaHsd female mice) that were 9-10 weeks at the start of training. Ten of these mice had a 6670 Hz tone as the safe and a 13340 Hz tone as the initial conditioned tone. The other 8 had the 13340 Hz tone as the safe and the 6670 Hz tone as the initial conditioned tone. The third replication, also run in Göttingen, used 7 mice that were 5-6 weeks at the start of training and heard a 7000 Hz tone as the safe and a 14000 Hz tone as the initial conditioned tone.

The differences between the experiment run in Jerusalem and those run in Göttingen include: the use of slightly younger (5-6 weeks at the beginning of the experiment) as well as older (9-10 weeks) animals, slight physical differences between the two apparatus, the use of roving intensities in the Göttingen replications, and the use of a low (7 kHz) and a high frequency (14 kHz) as both safe and conditioned in different groups. In addition the habituation phase lasted 3 days, instead of 7, and during the conditioning phases conditioned visits appeared at random intervals for a given mouse. Other than that the training protocol was identical to that used in the Jerusalem experiment (see main text).

To implement the intensity roving, the amplitude was roved around 67 dB. Roving yielded a variation in intensity for each frequency that was larger (range: 6 dB) than the differences in tone intensity at different positions within the corner when the same tone was played at a fixed nominal intensity (range: 4 dB), and also larger than the difference in intensity of tones with different frequencies (range: 4 dB, depending on frequencies and positions in the corner). This resulted in an overall amplitude variation of about 10 dB for each tone, with large overlap between the two tones. In consequence, the mice could not use intensity cues to discriminate between the two tones.

The same pattern of results as that obtained in Jerusalem and presented in the main text and Figure 3 was obtained in the replication experiments in Göttingen. Figure S1a represents the mean daily performance of animals that were 9 week old at the beginning of training and, therefore, 10 weeks old when conditioning began. Half the animals in this group were trained with a safe tone of 6670 Hz tone and a conditioned tone of 13340 Hz tone, while the other half were trained with the reverse assignment. There was no difference between the two groups. A 3-way ANOVA on the performance over the first 7 days of conditioning (day x training frequency x safe *vs* conditioned tone) revealed an effect of day (F(7,263)=2.23; p=0.03), no effect of training frequency (F(1,263)=1.74; p=0.18), and a strong effect of safe *vs* conditioned tone (F(1,263)=1139.42; p<0.01). There was a significant interaction between day and safe *vs* conditioned tone (F(7,263)=5.38; p<0.01) and between training frequency and safe *vs* conditioned tone (F(1,263)=6.15; p<0.01), but not between day and training frequency (F(1,263)=6.15; p=0.64). Therefore, the data from mice conditioned to 13340 Hz and to 6670 Hz were averaged together. Already on day 1, there was no effect of training frequency (F(1,33)=0.07, p=0.80) but a strong effect of the behavioural meaning of the tone (F(1,33)=22.2, p<0.01, training frequency x safe *vs* conditioned tone). Learning occurred, as in the main experiment, during the few trials following the first experience of an air-puff (Figure S1b). In Figure S1b, trial 1 is the first conditioned trial in which each mouse nose-poked and received an air-puff. Thus, mice learned to avoid nose-pokes in conditioned trials after experiencing a single air-puff. When considering the conditioned trials by the order in which they were presented, independently of whether the animal nose-poked or not, eight out of eighteen mice nose-poked in the first presentation of the conditioned tone and received an air-puff in consequence. By the 3^rd^ presentation, only 3 mice nose-poked.

In other respects as well, the pattern of performance was very similar to that of the main experiment. For example, the percentage of safe visits without nose-pokes was between 20-40%, a general finding in all the replications reported in this paper, as well as in our further studies with the same apparatus (unpublished data). Also, while during the habituation and safe phases the number of nose-pokes could vary quite widely, during the conditioning, most of the conditioned visits had no nose-pokes (Figure S1c). The one aspect of behaviour that differed slightly between Jerusalem and Göttingen was the duration of short visits with no nose-pokes. While in Jerusalem, the bulk of short visits without nose-pokes lasted 1 second or less, in Göttingen these visits were typically longer, 3 seconds or less (Figure S1d).

Figures S1e and f show the performance of mice that were trained in Göttingen but started the training at the age of 5-6 weeks. Their performance was similar to that of mice trained in the other replications. In this case, learning was a bit slower, with about half the mice requiring two air-puffs before learning to avoid nose-pokes during conditioned visits.

**Just noticeable differences (JNDs)**

The frequencies used for the JND phase run in Göttingen vary slightly with respect to those used in Jerusalem. See Table S1 for details. Once the smallest ΔF was tested, a final testing with a ∆f of 100% was run to ensure that the mice remained under stimulus control.

The two replications of the discrimination experiment in Göttingen reproduced the general pattern of results of the experiment run in Jerusalem. Figure S3a illustrates the behaviour of the mice that started the discrimination training at the age of 9 weeks. The first 3 data points are the mean across groups of the last 3 data points shown in Figure S1a, before the progressive reduction in ΔF. The gap in the graph represents a night in which the training program malfunctioned and the mice were not trained. When ΔF reached 4%, the performance in the conditioned visits was practically indistinguishable from that in the safe visits. A 3-way ANOVA on group x ΔFs x safe *vs* conditioned tone revealed a strong effect of group (F(1,695)=131.1, p<0.01), a strong effect of ΔF (F(7,695)=11.9, p<0.01), a strong effect of tone (F(1,695)=339, p<0.01), a group x tone interaction (F(1,695)=6, p<0.02), a ΔF x tone interaction (F(7,695)=27.21, p<0.01), but no group x ΔF interaction (F(7,695)=1.6, p=0.13). The group difference resulted from a difference in performance between the group that had the 6670 Hz tone as safe and the group that had the 13340Hz tone as safe. The first had high avoidance of the conditioned tone but also a high level of safe visits without nose-pokes (50% of the safe visits). The second group had a less impressive percentage of hits but also a smaller percentage of false alarms (about 40%). The pattern of results in both groups was, however, very similar and statistical analysis of the separated groups yields the same conclusions (data not shown). At a ΔF of 4% a 2-way ANOVA on day x safe *vs* conditioned tone revealed no effect of day (F(1,69)=0, p=0.96) and an effect of tone (F(1,69)=13, p<0.01). At a ΔF of 6% a 2-way ANOVA on day x safe *vs* conditioned tone revealed no effect of day (F(1,69)=0, p=0.99) and also no effect of tone (F(1,69)=3.7, p=0.06). Good discrimination was re-established at the end of training when the ΔF was set back to 100%.

We calculated d’ in the same way as in the main paper, with and without including the short visits (<3s, due to the difference in the distribution of visit lengths between Jerusalem and Göttingen), and found that a mean d’ of around 1 was obtained for ΔF of 10% when short visits were not included in the analysis. When all the visits were included, 44% of mice had a d' above 1 for ΔF of 10%. When short visits were removed, 68% of the mice had a d' above 1 ΔF of 10%.

As in the previous experiment, mice learned to generalize across frequencies and avoided novel tones already on their first presentation. This is illustrated in Figure S3c, showing the performance on conditioned trials followed by the performance on the subsequent 4 safe trials, using the same format as in Fig. 4c of the main text. It is clear that mice avoided nose-poking already on the first conditioned trials with 6 % <= ΔF <= 40 %.

Figure S3d-f show the same pattern of results for the animals that began training at 5-6 weeks of age. Focusing on Figure S3d, a 2-way ANOVA on ΔFs x safe *vs* conditioned tone revealed a strong effect of ΔF (F(4,200)=21, p<0.01), a strong effect of tone (F(1,200)=298, p<0.01), and an interaction (F(4,200)=18, p<0.01). At a ΔF of 7% a 2-way ANOVA on day x safe *vs* conditioned tone revealed no effect of day (F(2,38)<1) and an effect of tone (F(1,38)=5.21, p=0.03). Figure S3f shows a similar pattern of results as Figure S3c.
